# "Nebulized lidocaine for intractable cough in hospice care: a comprehensive review of efficacy, safety, and future perspectives"

**DOI:** 10.1186/s12904-025-01752-z

**Published:** 2025-04-30

**Authors:** Jumei Pan, Akhtar Ali Khan, Wenkai Yu, Lei Rui

**Affiliations:** 1 Department of Hospice Care of Linfen Road Community Health Care Center, Jing An District, Shanghai, 200435 China; 2 Department of Orthopedics, Tongji Hospital, Tongji Medical College, Huazhong University of Science and Technology, Wuhan, Hubei 430030 China

**Keywords:** Nebulized lidocaine, Intractable cough, Hospice care, Palliative care, Cough suppression, Safety

## Abstract

**Background and Objective:**

Intractable cough, affecting 10–50% of terminally ill patients, significantly impairs quality of life. Conventional therapies often fail due to dose-limiting side effects or inadequate efficacy, necessitating alternative treatments. This review evaluates the efficacy, safety, and clinical applicability of nebulized lidocaine for managing intractable cough in hospice care.

**Methods:**

A systematic literature search (1973–2023) across PubMed, MEDLINE, Embase, and Cochrane Library identified studies on nebulized lidocaine in hospice or palliative populations. Inclusion criteria the Cochrane Risk of Bias Tool and Newcastle–Ottawa Scale. Data on cough reduction, side effects, and dosing were synthesized thematically.

**Results:**

Among 265 screened studies, 58 met inclusion criteria. Nebulized lidocaine (1–4%) demonstrated rapid cough suppression (within 15 min) in 70% of cancer patients, with effects lasting 2–4 h. Mild side effects, including oropharyngeal numbness (15%) and bitter taste (10%), were transient. However, 25% of asthmatic patients experienced bronchoconstriction (forced expiratory volume in 1 s FEV1FEV1 decline ≥ 15%), resolving with bronchodilators. Lidocaine reduced opioid reliance and improved comfort in 80% of cases. Variability in efficacy was noted, with limited benefits in severe chronic obstructive pulmonary disease (COPD) with acute respiratory failure.

**Conclusion:**

Nebulized lidocaine offers a safe, non-invasive option for intractable cough in hospice care, minimizing systemic side effects. Its rapid action and compatibility with opioid-sparing regimens enhance palliative outcomes. However, cautious use is warranted in asthma and (COPD) due to bronchoconstriction risks. Future research should prioritize standardized dosing, long-term safety, and *Randomized controlled trials(*RCTs in diverse hospice populations.

## Introduction

Intractable cough, defined as a persistent and refractory cough unresponsive to conventional therapies, is a debilitating symptom affecting 10–50% of terminally ill patients, particularly those with advanced cancer or chronic respiratory diseases [1, 2]. In hospice populations, as many as 60% of lung cancer patients experience severe cough, often due to tumor invasion, airway compression, or pleural effusions [3, 4]. Similarly, patients with interstitial lung diseases (ILDs), including idiopathic pulmonary fibrosis (IPF), report cough as a dominant symptom, with prevalence rates ranging from 59 to 100% [5, 6]. Chronic cough is also prevalent in non-cancer conditions, such as connective tissue disease-related ILD and post-COVID- 19 interstitial lung disease, where mechanical distortion of airways and neuropathic pathways contribute to the persistence of symptoms [7, 8].

The impact of intractable cough on quality of life is profound, exacerbating physical and psychological distress, leading to fatigue, insomnia, and social isolation [9, 10]. In cancer patients, cough is frequently accompanied by dyspnea, pain, and vomiting, which collectively reduce the ability to engage in meaningful end-of-life interactions [2]. The psychological burden is significant, as anxiety and depression often accompany uncontrolled symptoms [11, 12]. For instance, 40% of people living with advanced lung disease and chronic cough report feeling'trapped'by their symptoms, resulting in withdrawal from family activities [13, 14]. In ILD patients, cough severity has been shown to correlate with lower health-related quality of life scores, highlighting the need for more effective, targeted interventions [8].

The management of intractable cough in people approaching the end of life presents substantial challenges. Standard therapies, including opioids, corticosteroids, and benzonatate, often fail due to dose-limiting side effects such as sedation and constipation, or inadequate efficacy [15, 16]. Opioids may reduce cough frequency by only 30–50% in cancer patients, but their use is often limited by significant side effects such as constipation, sedation, opioid toxicity, and opioid-induced hyperalgesia [17, 18]. Anticholinergics, conversely, may exacerbate dry mouth [19]. Non-pharmacological interventions, such as bronchoscopic stenting or radiation, are invasive or impractical in hospice settings [20]. Though compounded medications may occasionally be effective, they lack standardized formulations [21]. Emerging therapies, such as P2X3 inhibitors, have shown promise but remain investigational, leaving a significant gap in available accessible, non-invasive options [8, 22].

Hospice care aims to maximize comfort and dignity through a holistic, multidisciplinary approach to symptom management, which includes addressing intractable cough using multimodal strategies. These may integrate pharmacological agents, psychosocial support, and patient education [23]. For example, 75% of palliative care teams use individualized plans designed to balance efficacy with minimal invasiveness [15, 24, 25]. Speech pathology interventions, such as cough suppression techniques, are increasingly recognized for their role in reducing laryngeal hypersensitivity. However, their application in terminally ill patients requires further study [26, 27].

Effective suppression of cough is critical in hospice care, as uncontrolled symptoms can hasten terminal decline [28–31]. Palliative care guidelines emphasize early intervention for symptoms like dyspnea and cough, recognizing their role in accelerating functional deterioration [32]. In advanced ILD patients, 80% require tailored regimens to address concurrent dyspnea and cough [5]. Neuromodulators, such as gabapentin, have shown partial efficacy, though their systemic side effects limit their use in frail populations [33, 34]. Lidocaine, a sodium channel blocker, inhibits neuronal excitability in sensory nerves, including airway C-fibers and rapidly adapting receptors (RARs), thus offering a potential mechanism for cough suppression [9, 35]. Its anti-inflammatory properties help reduce airway hyperresponsiveness by suppressing cytokine release and neurogenic inflammation, positioning it as a promising candidate for managing intractable cough [34, 36]. Nebulized formulations of lidocaine deliver localized anesthesia to the tracheobronchial tree, minimizing systemic absorption and toxicity [9]. Preclinical studies indicate that silencing nociceptor neurons with lidocaine analogs reduces allergic airway inflammation, which highlights its dual role in modulating both immune and neural pathways [37, 38].

Nebulized lidocaine has demonstrated efficacy in reducing cough intensity in both chronic refractory and cancer-related coughs. For instance, a pilot study reported that 70% of cancer patients achieved significant symptom relief with nebulized lidocaine administered twice daily [9, 37, 39, 40]. Additionally, topical pharyngeal lidocaine has been shown to reduce respiratory adverse events during procedures, further suggesting its broader applicability in palliative care settings [41–43].

Lidocaine’s versatility extends beyond cough management. It is used in the management of neuropathic pain and airway hypersensitivity. For instance, intravenous lidocaine has been shown to reduce postherpetic neuralgia pain by 60% in patients with advanced cancer receiving palliative care [35, 44]. Its anti-inflammatory effects have also been explored in the context of COVID- 19-related hyperinflammation, though clinical evidence in this area remains preliminary [45]. These properties underscore lidocaine's potential for multimodal symptom control in hospice care [16, 46]. In hospice care, nebulized lidocaine is ideal due to its rapid onset, minimal side effects, and compatibility with opioid-sparing regimens [9, 28]. For bedbound patients, its non-invasive administration avoids the risks of sedation or aspiration associated with systemic therapies [10, 29]. A retrospective analysis of 34 cancer patients found that 71% experienced significant cough reduction with lidocaine, although larger trials are needed to confirm these findings [10, 47]. Moreover, lidocaine’s safety profile in pediatric populations, demonstrated through its use in laparoscopic procedures, supports its adaptability across various age groups [48, 49].While the existing literature remains limited, there is promising evidence regarding the use of lidocaine. A randomized trial of TRPV4 inhibitors highlighted the challenges of cough-specific therapies and the need for targeted treatments like lidocaine [50]. In contrast, studies on gabapentin and paroxetine demonstrate partial efficacy, but their systemic side effects limit their utility in frailer populations [34, 51, 52]. Emerging research on P2X3 inhibitors and speech therapy combinations offers promising new directions, yet lidocaine’s immediacy and accessibility make it a pragmatic choice in hospice settings [29, 33].

This review evaluates the safety, efficacy, and practicality of nebulized lidocaine for managing intractable cough in hospice patients, synthesizing evidence from palliative, respiratory, and pharmacological studies. By addressing gaps in current research—such as optimal dosing, long-term tolerability, and patient selection—the review aims to inform clinical practice and advocate for standardized protocols in end-of-life cough management. Ultimately, improving cough control aligns with the ethical principles of autonomy and beneficence, ensuring that patients experience dignity in their final days [53].

## Methods and data extraction

This review aimed to systematically explore the scientific literature on using nebulized lidocaine to manage intractable cough in terminally ill patients receiving hospice care. The methodology was designed to comprehensively and objectively synthesize the available evidence while maintaining scientific rigor. A systematic search was carried out across multiple electronic databases, including PubMed, MEDLINE, Embase, and the Cochrane Library, covering studies published from 1973 to 2023. The search terms combined keywords like"nebulized lidocaine","intractable cough","terminal illness","hospice care","palliative care","chronic cough", and"lidocaine efficacy". Boolean operators (AND, OR) were used to refine the search results. Manual searches of reference lists from relevant articles and reviews were also conducted to ensure no relevant studies were missed. Studies were included if they met specific criteria. The population was restricted to terminally ill patients or those with advanced—stage diseases (e.g., COPD, pulmonary fibrosis, cancer) experiencing intractable cough. The intervention had to involve nebulized lidocaine, either as a primary or secondary treatment for cough management. Studies also needed to report on the efficacy, safety, or side effects of nebulized lidocaine in reducing cough severity or frequency. Study designs included RCTs, observational studies, case reports, and case series. Studies focusing on non—nebulized forms of lidocaine (e.g., intravenous, epidural) or those not related to cough management in terminally ill or hospice care populations were excluded.

Data from eligible studies were extracted using a standardized template. This template included details such as study author(s), publication year, study design, duration, number of participants, demographic characteristics, disease/condition and its stage, lidocaine dosage, administration method, frequency, key findings on cough suppression, patient comfort, quality of life, reported outcomes (efficacy, adverse effects, patient tolerance), and side effects. The extracted data were then synthesized thematically, concentrating on the efficacy of nebulized lidocaine in reducing cough severity, its impact on patient comfort, and any reported side effects or limitations. Studies were grouped by disease condition to identify patterns and differences in outcomes among different patient populations.

**Fig. 1:**
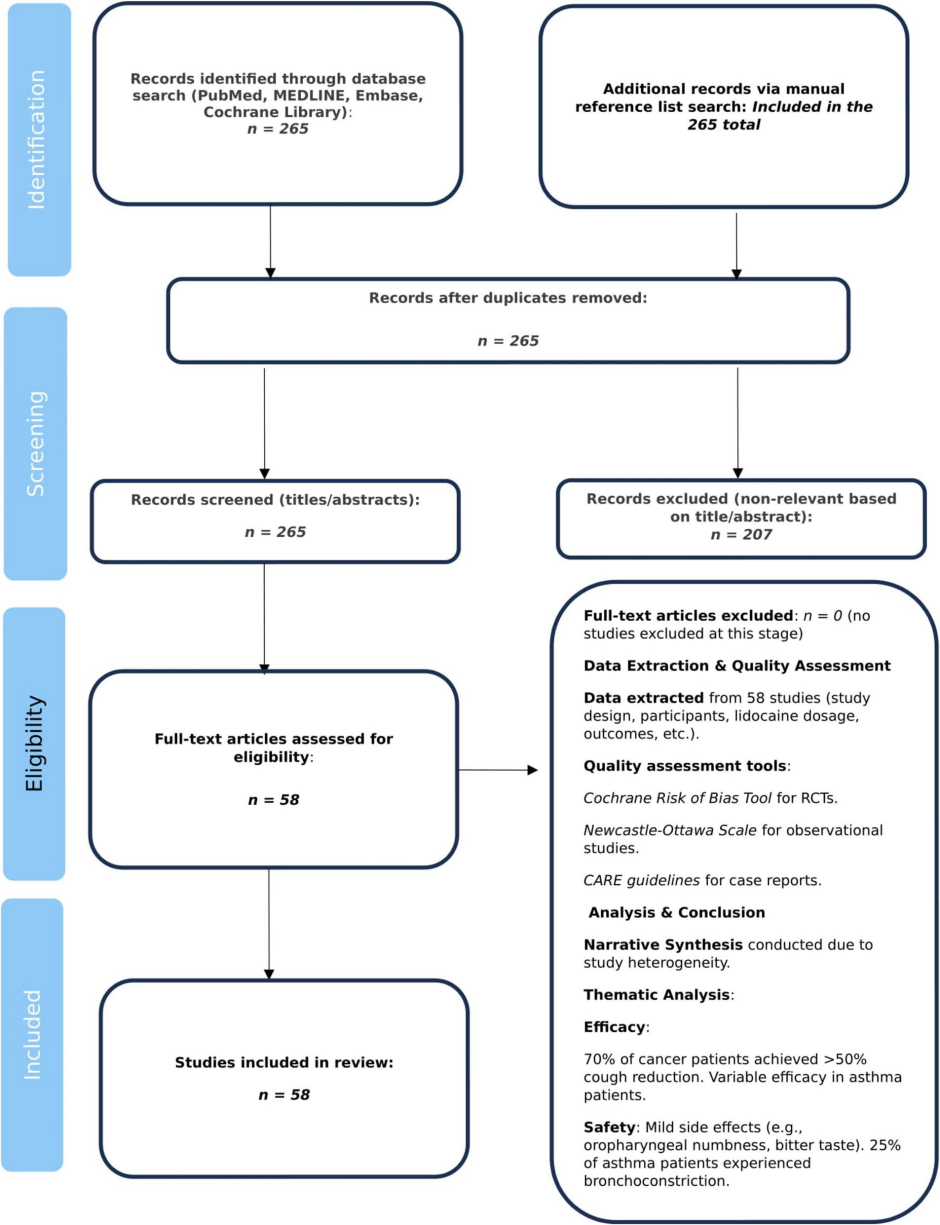
Study flow chart

The methodological quality of included studies was evaluated using appropriate tools for each study design. RCTs were assessed with the Cochrane Risk of Bias Tool, observational studies with the Newcastle—Ottawa Scale, and case reports with the CARE guidelines. This quality assessment enabled the findings to be interpreted in the context of each study's strengths and weaknesses. Due to the heterogeneity of study designs and patient populations, a meta—analysis was not possible. Instead, a narrative synthesis approach was used to summarize the findings. The results were organized into thematic categories, including evidence for the role of nebulized lidocaine in cough suppression and symptom relief. Reported side effects, such as oropharyngeal numbness, bitter taste, or bronchoconstriction, and their implications for use in hospice care were also considered. Finally, practical aspects of using nebulized lidocaine in terminally ill patients, such as dosing, frequency, and patient selection, were addressed. This review adhered to ethical guidelines for systematic reviews, ensuring transparency, reproducibility, and no data manipulation. Table 1. Figures 1. and 2.

**Table 1:** Summary of Studies on Lidocaine usage for Cough in respiratory patients

Year	Study Author(s)	Study Design/ duration	Number of participants	Disease/Condition	Stage of Disease	Lidocaine Dosage/Method	Key Findings	Outcome/Impact	Conclusion	Side effects
1979	J. Savoy, S. Dhingra, N. R. Anthonisen [54]	Observational, cross-sectional	10 patients (pulmonary fibrosis), 7 controls.)	Pulmonary Fibrosis	Advanced (End-Stage)	Lignocaine airway anesthesia (tested by cough response to citric acid inhalation)	- P0.1, f1, and VT/Ti were greater in patients with pulmonary fibrosis compared to controls.			
1996	Hunt, Swedlund, Gleich [55]	Prospective open study	20 (18 women, 2 men)	Severe Asthma	Severe, glucocorticoid-dependent	40 to 160 mg nebulized, 4 times daily	13 patients discontinued oral glucocorticoids; 4 reduced glucocorticoid dosage; 3 had no response.	65% of patients (13 out of 20) were able to discontinue oral glucocorticoids. 20% (4 out of 20) reduced glucocorticoid dose.	Nebulized lidocaine allows reduction or elimination of oral glucocorticoid therapy.	No specific side effects mentioned.
1996	von Schönfeld et al. [56]	Observational study	27 (7 with quantitative liver function tests)	Alpha-1-antitrypsin deficiency (Pi ZZ), Pulmonary Emphysema	Severe	Not specified, but lidocaine metabolism was measured (half-life)	- Liver function tests mostly normal in patients. - Mild liver enzyme elevation in 10 patients. - Lidocaine half-life was prolonged in 4 out of 7 patients.	- Lidocaine metabolism (measured by lidocaine half-life) was impaired in a subset of patients (4/7). - Liver function tests normal in the majority of patients.	No significant clinical liver disease or impairment in most patients. Lidocaine metabolism was impaired in some patients, but no statistically significant liver dysfunction in the group overall.	
1999	H. Morisaki, R. Serita, Y. Innami, Y. Kotake, J. Takeda [57]	Retrospective study, single-center	13	Emphysema	Severe (End-stage)	Lidocaine administered for ventricular arrhythmias (method not specified)	- 13 patients with severe emphysema undergoing thoracic surgery			
1999	M L Decco, T A Neeno, L W Hunt, E J O'Connell, J W Yunginger, M I Sachs [58]	Open-label pilot study, duration: 7 to 16 months (mean: 11.2 months)	6	Severe Asthma (in children)	Severe, steroid-dependent asthma	Nebulized lidocaine 0.8 mg/kg/dose to 2.5 mg/kg/dose, 3 to 4 times daily (t.i.d to q.i.d)	Five out of six patients discontinued oral glucocorticoids after a mean of 3.4 months	5 of 6 patients completely discontinued oral glucocorticoids (mean 3.4 months)	Lidocaine may serve as a non-toxic steroid alternative in steroid-dependent asthma	No specific side effects mentioned.
2001	K. Nozaki, A. Endou, K. Sakurai, O. Takahata, H. Iwasaki [59]	Case Report (Duration not specified)	1 (76-year-old male)	Giant Bulla and Liver Cirrhosis	End-stage lung disease (giant bulla) with liver cirrhosis	Epidural analgesia with 2% lidocaine (continuous injection)	-Laryngeal mask airway (LMA) and epidural analgesia were used for anesthesia. -2% lidocaine was injected into the epidural space continuously to achieve abdominal muscle relaxation and pain management.	No neurological abnormalities or rupture of the giant bulla were observed post-surgery.	The combination of LMA and epidural lidocaine reduced the risk of giant bulla rupture during surgery and provided effective pain management.	No specific side effects mentioned.
2001	T. Kimura, T. Mizutani [60]	Case Report/Duration: 155 days (Postoperative period)	1	Unilateral Pulmonary Fibrosis, Postoperative Complications	Advanced lung disease (postoperative, progressive fibrosis)	Epidural Lidocaine (no specific dosage reported)	Rare case of unilateral pulmonary fibrosis following ipsilateral single-lung ventilation and anesthesia	The left lung developed honeycomb patterns and fibrosis, with organized pneumonia in the right lung. No autopsy performed.		The patient died of respiratory failure. No clear correlation with lidocaine was found, but other mechanical factors likely contributed. Lidocaine administration (epidural) is unlikely to be the sole cause.
2005	C-F Chong, C-C Chen, H-P Ma, Y-C Wu, Y-C Chen, T-L Wang [11]	Prospective comparison study (Tertiary emergency department)	127 (62 received lidocaine, 65 received bronchodilator)	Chronic Obstructive Pulmonary Disease (COPD)	Intractable cough in COPD patients	Nebulized lidocaine	Lidocaine and bronchodilator both effectively suppressed cough in COPD patients. No significant difference in efficacy between treatments.	Cough severity score significantly reduced 1 hour after treatment (both groups). No significant difference in efficacy between lidocaine and bronchodilator.	Both lidocaine and bronchodilator inhalation are equally effective for short-term cough suppression in COPD patients.	Common mild side effects: lidocaine group (oropharyngeal numbness, bitter taste); bronchodilator group (tremor, palpitation). No serious adverse events.
2006	Shigekazu Sugino, Keiichi Omote, Mikito Kawamata, Akiyoshi Namiki [61]	Case report	1	Chronic Obstructive Pulmonary Disease (COPD)	Severe COPD	5 ml of 1.0% lidocaine injected intradermally in the right elbow for catheter sheath insertion	The patient underwent elective endovascular repair for an aortic abdominal aneurysm without intubation. Spinal anesthesia and local anesthesia were used. Lidocaine was used as part of the local anesthesia for the catheter insertion.	The procedure was completed uneventfully, and the patient did not experience postoperative respiratory failure, despite the severe COPD.	Spinal anesthesia combined with local anesthesia (including lidocaine) was deemed effective for managing this procedure in a patient with severe COPD. The patient experienced no major complications.	No specific side effects mentioned.
2006	Keiko Saito, Nagato Sato, Nobutaka Shimono, Kouichi Hagiwara, Minoru Kanazawa, Makoto Nagata [62]	Case report (no specific duration mentioned)	1	Severe Asthma	Severe, uncontrolled asthma during pregnancy	Inhalational lidocaine 40-100mg via ultrasonic nebulizer, 5 times/day	Inhalational lidocaine dramatically improved symptoms (wheezing, cough, hypoxemia) and allowed reduction of systemic corticosteroids.	Patient's asthma symptoms significantly improved, leading to the possibility of reducing systemic corticosteroid use.	Inhalational lidocaine may be a useful supplementary treatment for refractory asthma, particularly in pregnant cases.	No specific side effects mentioned.
2007	Pyng Lee, Henri G. Colt [63]	Case series (single procedure)	5 patients	Pneumothorax, Severe Chronic Obstructive Pulmonary Disease (COPD)	Advanced (end-stage COPD with pneumothorax)	250 mg Lidocaine via spray catheter before talc poudrage	Lidocaine administered via spray catheter was effective for pain control before talc poudrage in patients with pneumothoraces and severe COPD.	Pain scores were 3, 2, and 2 on postoperative days 0, 1, and 2, respectively. No complications or 30-day mortality.	Lidocaine via spray catheter provided effective pain relief.	No specific side effects mentioned.
2009	Miroslava Kapala, Sarkis Meterissian, Thomas Schricker [64]	Case Report	1	Severe COPD, Obstructive Sleep Apnea (OSA)	Severe/Advanced COPD and OSA	Intrathecal: 3.5 mL isobaric bupivacaine 0.5% with 100 µg epinephrine and 200 µg morphine; Epidural: 60 mg bupivacaine, 200 mg lidocaine	Combined spinal-epidural anesthesia with BiPAP for an elective sigmoid resection in a patient with severe COPD and OSA; perioperative BiPAP and continuous epidural infusion for pain control.	Excellent pain relief with continuous epidural infusion of local anesthetics; no adverse respiratory events.	Combined spinal-epidural anesthesia and perioperative BiPAP contributed to an uncomplicated postoperative course.	No specific side effects mentioned.
2010	A. Molassiotis, G. Bryan, A. Caress, C. Bailey, J. Smith [65]	Systematic Review of Trials	1177 trials identified, 75 included	Respiratory and Non-respiratory Diseases (excluding cancer)	Chronic Cough	Lidocaine was evaluated in some studies	Positive results were seen with lidocaine among other treatments	Evidence of efficacy		No specific side effects mentioned.
2012	K. Stuart-Smith [66]	Case Report	1	Severe Cardiac and Respiratory Disease (End-stage pulmonary disease)	Severe/End-stage (Cardiac and Respiratory Dysfunction)	Lidocaine 1% (20 ml) and Bupivacaine 0.5% (20 ml) for Transversus Abdominis Plane Block	The hemiarthroplasty was performed successfully with transversus abdominis plane block as the main anaesthetic technique, avoiding general or spinal anaesthesia risks in a patient with severe cardiorespiratory dysfunction.	The patient remained alert and comfortable, with no need for further analgesia during the procedure. First opioid administration occurred 12 hours post-surgery.	Surgery completed without complications. The patient succumbed to respiratory disease 6 months post-surgery.	No specific side effects mentioned.
2014	Naomi Ono, Nobuyasu Komasawa, Shoko Nakano, Shinichi Tatsumi, Kenta Nakao, Toshiaki Minami [67]	Case report (Duration not specified)	1	Emphysema with Pneumothorax due to Metastatic Lung Cancer	Advanced/End-stage (Multiple metastatic cancer, emphysema, pneumothorax)	Lidocaine via epidural anesthesia at T8-9 (in combination with dexmedetomidine)	The patient underwent successful one-lung ventilation with spontaneous breathing, using dexmedetomidine and regional anesthesia (lidocaine). No complications from the procedure.	No complications reported; stable throughout the procedure	One-lung ventilation under dexmedetomidine sedation with lidocaine as regional anesthesia effectively prevented barotrauma in this patient with metastatic lung cancer and emphysema	No specific side effects mentioned.
2014	Casilda Olveira, Ana Muñoz, Adolfo Domenech [68]	Review (No direct experimental data provided)	Not applicable	Various pulmonary diseases (e.g., COPD, fibrosis)	End-stage or advanced lung disease (focused on adults)	Nebulized therapy (e.g., lidocaine included in discussion)	Nebulized drugs are recommended for patients requiring high doses of bronchodilators, antibiotics, mucolytics, and other treatments, especially when other inhalation methods are not feasible. Technological advancements in devices enhance pulmonary drug deposition and reduce treatment time.	Highlights efficacy in optimized drug deposition.	Nebulized therapy has fewer systemic side effects compared to systemic administration but does not provide specific statistical data on side effects.	Implies fewer systemic side effects with nebulized therapy compared to systemic administration.
2017	Adrian Sultana, David Torres, Roman Schumann [69]	Review/Discussion of Various Case Reports & Prospective Studies	Not specified	Chronic Obstructive Pulmonary Disease (COPD), Obesity, Sleep Apnoea, Complex Regional Pain Syndromes, Opioid Addiction, Cancer Surgery	Advanced stages in some cases, depending on the disease	Intravenous lignocaine (Lidocaine) used in opioid-free anaesthesia techniques	Lignocaine used for sympatholysis (reduction of sympathetic nervous system activity) and co-analgesia	The review found benefits in using opioid-free techniques, including improved outcomes for patients with COPD and obesity. Specifically, lignocaine helped reduce the need for opioids and improved perioperative analgesia.	No specific statistical results provided in the abstract, but the benefits of opioid-free anaesthesia were noted for reducing opioid-related complications	Side effects or adverse reactions to lignocaine or opioid-free anaesthesia were not discussed in detail. However, general concerns about the avoidance of opioids (e.g., in COPD patients) may be a consideration for managing respiratory function.
2023	Yongbin Wang, Chang Feng, Jia Fu, Dongyi Liu [70]	Randomized Controlled Trial (RCT), Duration not specified	60 patients	Chronic Obstructive Pulmonary Disease (COPD)	Severe COPD	2 mL of 2% lidocaine for bilateral internal branch of the superior laryngeal nerve block	- Shortened intubation time in lidocaine group. - Significantly fewer adverse reactions in lidocaine group. - Higher comfort score in lidocaine group. - Better haemodynamic stability and reduced stress response (lower MAP, HR, NE, and AD in lidocaine group) .	- Significant reduction in intubation time (*P*<0.01). - Significantly fewer adverse events and better comfort score (*P*<0.01). - Haemodynamic stability and stress markers significantly lower in lidocaine group (*P*<0.05).	Ultrasound-guided internal branch of superior laryngeal nerve block using lidocaine reduces intubation time, adverse reactions, and improves comfort in severe COPD patients undergoing awake fibreoptic nasotracheal intubation.	No specific side effects mentioned.
1973	Frank Rodriguez-Martinez, Armand V Mascia, Robert B Mellins [71]	Experimental, cross-sectional study	8 children with chronic asthma and 9 controls	Asthma	Chronic asthma	Topical lidocaine spray applied to the nose and pharynx	- Cold-induced increase in airway resistance in untreated asthmatic children	- Significant decrease in FEV1, MVV, V50, and V25 at low temperature in untreated asthmatic children (*P* < 0.01) - No significant change in test results after lidocaine treatment (*P* > 0.05)	Cold-sensitive receptors are present in the upper airways, and cold-induced bronchoconstriction is reflexive	No specific side effects mentioned.
1977	Earle B. Weiss, M.D., F.C.C.P. & Avinash V. Patwardhan, M.D. [72]	Observational study	22 patients	Bronchial Asthma	Stable asthma	Aerosol: 40 mg and 100 mg doses; Intravenous: 1 mg/kg body weight	Initial response: ~20% fall in expiratory airflow rates within 5 minutes of aerosol administration. Bimodal response: Group 1 showed continued reduction in airflow; Group 2 showed improvement in airway resistance.	Aerosol 100 mg dose: Group 1 saw maximal decrease in FVC (−24.6%), FEV1.0 (−38.0%), MMEFR (−42.6%). Group 2 saw significant improvement in FVC (+11.8%), FEV1.0 (+25.2%), MMEFR (+41.0%). Intravenous lidocaine: Mild bronchodilator effect.	Aerosol lidocaine produced a variable response: a significant fall in airway flow for some and improvement in others. Intravenous lidocaine was only mildly effective as a bronchodilator.	No specific side effects mentioned, brief initial bronchoconstriction was noted.
1979	J E Fish, V I Peterman [73]	Experimental, cross-sectional study (duration not specified)	8 asthmatic subjects	Asthma	Not specified (general asthma subjects)	Inhaled 2 cm³ of lidocaine (4%)	- Significant fall in FEV1 (23.4 ± 4.8%) and SGaw (64.1 ± 3.8%) after inhalation of lidocaine (*p* < 0.001) - Bronchoconstrictor effects were reversed with aerosolized atropine or isoproterenol.	FEV1: -23.4 ± 4.8%, SGaw: -64.1 ± 3.8% (statistically significant, *p* < 0.001).	Inhaled lidocaine causes bronchoconstriction in asthmatic subjects, which can be prevented or reduced by bronchodilators (aerosolized atropine or isoproterenol).	Reflex-mediated bronchoconstriction.
1980	P.L. Enright, J.F. McNally, J.F. Souhrada [74]	Experimental (with treadmill exercise testing)	Not specified	Bronchial Asthma	Not specified	4% Lidocaine aerosol, delivered during the last third of inspiration	Lidocaine blocked exercise-induced bronchoconstriction (EIB), as measured by multiple pulmonary parameters (FEV1, FEF25-75%, Vmax70% TLC, SGaw). Decreased minute ventilation (VE) during exercise.	EIB was significantly inhibited, and VE during exercise decreased (*p* < 0.01).	Local anesthesia of the upper and large airways significantly inhibits EIB and decreases VE during moderate exercise.	No specific side effects mentioned, but brief absence of cough and gag reflexes for 15-20 minutes after treatment.
1980	C H Fanta, R H Ingram Jr, E R McFadden Jr [75]	Experimental (Eucapnic hyperventilation challenge)	10	Exercise-induced asthma (EIA)	N/A	Oropharyngeal spray of lidocaine vs. water	- No change in pulmonary mechanics at rest after lidocaine or water spray. - Both lidocaine and water followed by significant reduction in pulmonary function after hyperventilation. - No difference between lidocaine and water.	Significant reduction in pulmonary mechanics observed after eucapnic hyperventilation in both conditions (lidocaine and water)	The study did not find evidence for "irritant-like" receptors in the posterior pharynx affecting airway cooling during exercise-induced asthma.	No specific side effects mentioned.
1982	W M Tullett, K R Patel, K E Berkin, J W Kerr [76]	Single-blind trial (duration not specified)	8	Exercise-induced asthma	Not specified	Inhaled lignocaine (48 mg)	No significant change in baseline FEV1 or MMFR after lignocaine. Ipratropium bromide caused bronchodilation, sodium cromoglycate had inhibitory effects, lignocaine had no significant effect.	Maximal percentage fall in FEV1 after exercise: saline (38.1%), lignocaine (34.5%), sodium cromoglycate (11.3%), ipratropium bromide (19.3%). Maximal fall in MMFR: saline (54.4%), lignocaine (52.9%), sodium cromoglycate (23.6%), ipratropium bromide (32.1%).	Sodium cromoglycate and ipratropium bromide had significant protective effects, while lignocaine did not. Local anaesthesia of sensory vagal receptors is not effective in preventing exercise-induced asthma.	No specific side effects mentioned.
1982	D Murciano, M Aubier, F Viau, S Bussi, J Milic-Emili, R Pariente, J P Derenne [77]	Observational study; no specified duration	14	Chronic Obstructive Pulmonary Disease (COPD)	Acute respiratory failure	Airway anesthesia by fiberoptic xylocaine from the larynx to subsegmental bronchi	1. Decrease in minute ventilation by 6 ± 1%.2. Respiratory frequency decreased by 14.5 ± 1%.3. Increased expiratory time.4. Increased tidal volume by 10.1 ± 0.6%.5. Arterial blood gas deterioration.	PaO2: 42 ± 3 mmHg (post-anesthesia) vs 48 ± 2 mmHg (control).PaCO2: 62 ± 3 mmHg (post-anesthesia) vs 54 ± 2 mmHg (control).	Activation of airway receptors contributes to rapid and shallow breathing in COPD patients during acute respiratory failure.Airway xylocaine anesthesia worsens blood gases and is contraindicated in these patients.	Worsened arterial blood gases: decrease in PaO2 and increase in PaCO2.
1983	H Downes, C A Hirshman, D A Leon [78]	Comparative study, experimental in dogs (Basenji-Greyhound), duration of aerosol administration: 10 minutes before citric acid challenge	Basenji-Greyhound dogs (specific number not mentioned in abstract)	Airway constriction (provoked by citric acid aerosols)	Airway constriction induced by citric acid challenge (not specified as a disease but more of an experimental provocation)	4% Lidocaine aerosol for 10 minutes immediately preceding citric acid challenge	- Lidocaine aerosols (4%) did not prevent citric acid-induced increases in pulmonary resistance or changes in dynamic compliance.- Other anesthetics (bupivacaine, hexylcaine, procaine) showed similar results.	- Pulmonary resistance increased by 3.3 +/- 0.8 cmH2O X 1(-1) X s in controls.- Lidocaine: 2.1 +/- 0.6 cmH2O X 1(-1) X s.- Bupivacaine: 3.2 +/- 1.3 cmH2O X 1(-1) X s.- Procaine: 3.3 +/- 1.0 cmH2O X 1(-1) X s.- Hexylcaine: 2.1 +/- 0.6 cmH2O X 1(-1) X s.	Local anesthetic aerosols, including lidocaine, were ineffective in preventing airway constriction provoked by non-reflex stimuli.	No specific side effects mentioned.
1984	W Hida, M Arai, C Shindoh, Y N Liu, H Sasaki, T Takishima [79]	Observational study on inspiratory flow rate and bronchomotor tone, no duration specified in the abstract	31 (9 normal subjects, 22 asthmatic subjects)	Asthma, Bronchoconstriction (methacholine-induced)	Asthmatic subjects with spontaneous airway narrowing; methacholine-induced bronchoconstriction	Lignocaine (Lidocaine) inhalation	- In normal subjects with methacholine-induced bronchoconstriction, rapid deep inspiration reduced resistance more than slow inspiration. - Asthmatic subjects showed increased resistance after deep inspiration, greater after rapid inspiration. - Methacholine-induced bronchoconstriction showed greater bronchodilatation with rapid deep inspiration. - Lignocaine inhalation attenuated both bronchoconstriction and bronchodilatation.	Not specified numerically in the abstract; however, Lignocaine reduced bronchomotor tone significantly with both rapid and slow inspirations.	- Deep inspiration affects bronchomotor tone, and its effects differ between normal and asthmatic subjects. - Lignocaine inhalation attenuates bronchoconstriction and bronchodilatation, suggesting a role of irritant and stretch receptors in bronchomotor tone regulation.	No specific side effects mentioned.
1986	A Van Meerhaeghe, M Bracamonte, R Willeput, R Sergysels [80]	Experimental study (unspecified duration)	16 (8 normal, 8 COPD patients)	Chronic obstructive pulmonary disease (COPD)	Eucapnic (stable) COPD, Normal	4% lidocaine aerosol (240 mg) delivered to upper and large airways	1. Lidocaine eliminated gag and cough reflexes in all subjects. 2. No significant changes in pulmonary function at baseline or during exercise. 3. No effect on O2 intake (VO2) or blood gases during exercise	No significant changes in respiratory variables during exercise, before or after lidocaine (*p* > 0.05)	Vagal upper and large airway receptors do not play a significant role in breathing pattern or ventilatory drive during exercise in either normal subjects or COPD patients.	No specific side effects mentioned.
1987	W.Y. Chen, H. Chai [81]	Experimental; 2 sessions of 10 min treadmill exercise	5	Exercise-induced asthma	Not specified	Inhaled aerosol lidocaine (1.5 mg/kg)	- Pulmonary function tests measured before, after lidocaine inhalation, and post-exercise.- Decreased FEV1 and FEF25-75% post-exercise in both control and lidocaine sessions.	- Control session: FEV1 61% and FEF25-75% 44% of baseline.- Lidocaine treatment: FEV1 54% and FEF25-75% 44% of baseline.- No significant difference between control and lidocaine.	Afferent nerves in the respiratory mucosa do not play a critical role in exercise-induced asthma development.	No specific side effects mentioned.
1989	M Söderberg, R Lundgren [82]	Experimental study; no duration mentioned	21 healthy non-smoking volunteers, 6 asthmatics (for additional tests)	Healthy volunteers, Asthma	Healthy, Asthmatic	Topical anesthesia with lignocaine	FFB did not alter airway responsiveness; atropine and lignocaine had no major influence on airway responsiveness	No statistical changes in airway responsiveness before or after FFB in both groups	Epithelial damage in the central airways induced by FFB is not sufficient to induce bronchial hyperresponsiveness	No specific side effects mentioned.
1989	L.G. McAlpine, N.C. Thomson [83]	Observational, cross-sectional (single-day study)	20	Asthma	Moderate/Stable	6 ml 4% topical lidocaine (Xylocaine 4%) inhaled	- No correlation between histamine airway responsiveness and lidocaine-induced bronchoconstriction. - 25% of patients showed >15% fall in FEV1, with a maximum of 42.1%. - No difference in response to lidocaine with or without preservative.	25% of asthmatic patients experienced significant bronchoconstriction (>15% fall in FEV1).Maximum fall was 42.1%.	Inhaled topical lidocaine can induce significant bronchoconstriction in asthmatic patients.The response is not related to histamine airway responsiveness or preservative content.	Bronchoconstriction (up to 42.1% fall in FEV1) in 25% of patients.
1989	N Caire, A Cartier, H Ghezzo, J L'Archevêque, J L Malo [84]	Randomized, single-blind, cross-over study with 4 visits (2 placebo, 2 active), max interval of 3 weeks	8	Asthma	Clinical steady state	40 mg inhaled lignocaine solution	No significant changes in FEV1 and PD20 after inhalation of lignocaine vs placebo	No significant changes in forced expiratory volume (FEV1) or PD20 after lignocaine inhalation (*p* > 0.05)	Inhaled lignocaine does not significantly alter bronchial hyperresponsiveness to cold dry air in asthmatic subjects	No specific side effects mentioned.
1990	G S Prakash, S K Sharma, J N Pande [85]	Single-blind study, Duration not specified	18	Chronic stable asthma	Stable asthma	4% Lidocaine inhalation	- V50 at 15 min significantly decreased (*p* < 0.05).- 8 out of 15 patients showed decreased airway resistance (Raw).- 7 out of 15 patients showed increased specific airway conductance (SGaw).- No significant change in pulmonary function after 30 min.	- V50 decrease in 8/18 patients.- V25 decrease in 6/15 patients.- FEF25-75 decrease in 5/15 patients after 15 min.- No significant effect after 30 min.	Lidocaine produces a small bronchodilatory effect on large airways and a bronchoconstrictor effect on small airways after 15 min of inhalation. The effect is not statistically significant but is safe for use in bronchoscopy for patients with asthma.	No specific side effects mentioned.
1991	S Nakai, Y Iikura, K Akimoto, K Shiraki [86]	Experimental (Intradermal and bronchial challenge tests)	Not specified	Bronchial Asthma, Allergic Disorders	Moderate to Severe Asthma	Lidocaine (concomitant use)	Substance P induced stronger cutaneous and bronchial reactions in children with moderate or severe asthma compared to controls. Lidocaine inhibited these reactions, including intradermal erythema, wheal reactions, and bronchial airflow limitations.	Significant inhibition of substance P-induced erythema, wheal reactions, and airflow limitations. Lidocaine showed effectiveness in histamine-induced erythema and house dust-induced wheal and erythema reactions.	Substance P plays a role in cutaneous and bronchial hypersensitivity in children with asthma. Lidocaine can inhibit substance P-induced reactions effectively.	No specific side effects mentioned.
1993	H K Makker, S T Holgate [87]	Clinical Trial, Duration not specified	11	Asthma	Not specified	Inhaled lidocaine	Hypertonic saline challenge induced bronchoconstriction in asthma. Atropine, ipratropium, and lidocaine increased PD20HS.	PD20HS increased 2.6 times with lidocaine. No significant effect on baseline FEV1.	Anticholinergic and local anesthetic drugs, including lidocaine, suggest neurogenic reflexes contribute to hypertonic saline-induced bronchoconstriction in asthma.	No specific side effects mentioned.
1997	A. A. Floreani, S. I. Rennard [88]	Experimental/Review (no clear duration)	Not specified	Asthma	Not specified	Inhaled or systemic	Lidocaine may play a role in asthma therapy through inhibition of neurogenic inflammation and possibly mast cell function.	No specific statistics provided in the abstract.	Lidocaine could be beneficial in treating asthma, particularly in reducing neurogenic inflammation and mast cell activity.	No specific side effects mentioned.
1998	T. W. Harrison, A. E. Tattersfield [89]	Randomized, double-blind, placebo-controlled study	20	Asthma	Mild to moderate asthma	Inhaled lignocaine 40 mg and 160 mg (single doses), saline placebo	Lignocaine caused an initial fall in FEV1 for all doses compared to placebo; no significant difference between treatments.	FEV1 change: 0.13, 0.19, and 0.231 for saline, 40 mg and 160 mg, respectively (*P* = 0.2). No significant effect on heart rate, blood pressure, or bronchial reactivity to methacholine.	Single doses of inhaled lignocaine are well tolerated in mild to moderate asthma; bronchoconstriction prevented with salbutamol pretreatment.	No specific side effects mentioned, no effect on heart rate, blood pressure, or bronchial reactivity.
2000	H. Groeben [90]	Observational study (exact duration not specified)	Not specified	Bronchial hyperreactivity	Not specified	Intravenous lidocaine: 1.5-2.0 mg/kg	High thoracic epidural anesthesia causes a slight decrease in vital capacity but does not increase airway resistance or bronchial reactivity. Local anesthetics decrease bronchial reactivity, mainly due to systemic lidocaine.	Intravenous lidocaine (1.5-2.0 mg/kg) shows an effect similar to salbutamol. An additive effect occurs when combined with salbutamol.	High thoracic epidural anesthesia can be safely used in patients with bronchial hyperreactivity. Intravenous lidocaine can be used prophylactically before airway instrumentation.	No specific side effects mentioned.Possible concerns about motor blockade and pulmonary sympathicolysis.
2000	A D Maslow, M M Regan, E Israel, A Darvish, M Mehrez, R Boughton, S H Loring [91]	Prospective, randomized, double-blind, placebo-controlled trial	110 total (60 for lidocaine vs placebo group, 50 for albuterol vs placebo group)	Asthma	Not specified (general asthma, likely stable or mild to moderate cases)	1.5 mg/kg intravenous lidocaine administered 3 minutes before tracheal intubation	- Lidocaine did not reduce airway reactivity post-intubation. - Albuterol significantly reduced airway resistance (RL) post-intubation.	- No significant difference in peak RL between lidocaine and placebo groups (8.2 vs 7.6 cm H2O.l-1.s-1). - Albuterol group had significantly lower peak RL (5.3 vs 8.9 cm H2O.l-1.s-1, *P* < 0.05). - Albuterol group had lower frequency of bronchoconstriction (1 of 25 vs 8 of 23, *P* < 0.05).	Inhaled albuterol blunted airway response to tracheal intubation in asthmatic patients, whereas intravenous lidocaine did not.	No specific side effects mentioned.
2000	E.L. Langmack, R.J. Martin, J. Pak, M. Kraft [92]	Prospective, observational study	51	Asthma (mild to moderate)	Mild to moderate asthma	600 ± 122 mg (8.2 ± 2.0 mg/kg) administered topically to the upper airway and tracheobronchial tree for bronchoscopy	No signs or symptoms of lidocaine toxicity. Serum lidocaine concentrations (SLC) ranged from 0.10-2.90 mg/L at time 1 and 0.50-3.20 mg/L at time 2.	No significant relationship between SLC and age, sex, weight, baseline FEV1, procedure length, or study protocol. Significant correlation with lidocaine dose: time 1 (*r* = 0.33, *p* = 0.021); time 2 (*r* = 0.33, *p* = 0.023).	600 mg (8.2 mg/kg) of lidocaine appears to be safe for mild to moderate asthmatics undergoing research bronchoscopy.	No specific side effects mentioned.
2000	H Groeben, T Grosswendt, M Silvanus, M Beste, J Peters [90]	Experimental study	15 mild asthmatic patients	Asthma	Mild asthma (responsive to histamine challenge)	Inhalation of lidocaine at 1%, 4%, and 10% concentrations, total doses of 0.5, 2.0, and 5.0 mg/kg	Lidocaine inhalation attenuates bronchial hyper-reactivity but causes airway irritation. Increasing concentration increases initial bronchoconstriction.	FEV1 decreased most at the highest dose (from 3.79 ± 0.15 to 3.60 ± 0.15, *P* = 0.0012). PC20 significantly increased after lidocaine inhalation (baseline: 6.1 ± 1.3 mg/mL to 18.3 ± 4.5 mg/mL with 10% dose). No significant difference in anaesthesia duration between 4% and 10% solutions.	The most effective dose for local anaesthesia and attenuation of bronchial hyper-reactivity with the least airway irritation is 2.0 mg/kg as a 4% solution.	Plasma concentrations were below toxic thresholds. Bronchoconstriction at higher doses. Airway irritation present but manageable.
2000	Maslow AD, Regan MM, Israel E, et al. [91]	Prospective randomized controlled trial	60	Asthma (status asthmaticus)	Acute status asthmaticus	1.5 mg/kg lidocaine or saline given 3 minutes before intubation	No significant difference in pulmonary resistance (8.2 vs 7.6 cm water, ns). Frequency of airway response (6/30 vs 5/27, ns)	No significant difference in airway response or pulmonary resistance	No evidence to support use of intravenous lignocaine as a pretreatment for intubation-induced bronchoconstriction in asthmatic patients	No specific side effects mentioned.
2004	Hunt LW, Frigas E, Butterfield JH, Kita H, Blomgren J, Dunnette SL, Offord KP, Gleich GJ [93]	Randomized, placebo-controlled study, 8 weeks	50 (25 lidocaine, 25 placebo)	Asthma (mild-to-moderate)	Mild-to-moderate asthma	Nebulized lidocaine 4%, 100 mg, 4 times daily	Improved FEV1, decreased nighttime awakenings, reduced bronchodilator use, lower blood eosinophil counts in the lidocaine group	FEV1 (*P* ≤ 0.001), Nighttime awakenings (*P* ≤ 0.02), Symptoms (*P* ≤ 0.01), Bronchodilator use (*P* ≤ 0.01), Blood eosinophils (*P* ≤ 0.02); placebo group showed worsened symptoms and increased eosinophils	Nebulized lidocaine was effective and safe in improving asthma symptoms and reducing glucocorticoid use in mild-to-moderate asthma	No specific side effects mentioned.
2007	Herng-Yu Sucie Chang, Alkis Togias, Robert H. Brown [94]	Observational Study (No Duration Mentioned)	15	Asthma	Not specified	IV Lidocaine infusion	-Significant decrease in FEV1 (7 +/- 2%, *P* = 0.006). - Small decrease in airway luminal diameter (-3 +/- 0.5%, *P* < 0.001). 3. Significant correlation between change in FEV1 and airway luminal diameter (r2 = 0.47, *P* = 0.01).	-Decrease in FEV1 (7%)- Significant narrowing of airway diameter (-3%)	Lidocaine increases airway tone and narrows the airways during infusion in asthmatic subjects. It does not reduce baseline airway tone.	No specific side effects mentioned.but the study suggests the need for monitoring airways due to potential airway narrowing during lidocaine infusion.
2007	Michael Adamzik, Harald Groeben, Ramin Farahani, Nils Lehmann, Juergen Peters [95]	Controlled Clinical Trial, Duration not specified	30	Asthma	Not specified	2 mg/kg IV for 5 min, followed by 3 mg/kg/h for 10 min	Lidocaine mitigates bronchoconstriction after intubation in patients with asthma.	Airway resistance decreased by 26% after lidocaine administration (*p* < 0.004), compared to a 38% increase with saline.	IV lidocaine mitigates bronchoconstriction in asthma patients post-intubation.	No specific side effects mentioned.
2008	Burches, Bobby R. Jr BS; Warner, David O. MD [96]	Case Report, October 2008	1 (17-month-old female)	Asthma (Mild Intermittent Asthma)	Mild Intermittent Asthma	IV lidocaine, 1.5 mg/kg (13 mg total)	Bronchospasm developed after IV lidocaine injection during anesthesia induction	Bronchospasm resolved in approximately 5 minutes without further interventions.	IV lidocaine may cause bronchospasm in pediatric patients with asthma, but it was transient and self-limited.	Bronchospasm, transient and self-limited, with no evidence of anaphylaxis or aspiration.
2008	Chih-Chuan Lin, Kuan Fu Chen, Chia-Pang Shih, Chen-June Seak, Kuang-Hung Hsu [97]	Observational, retrospective cohort study	149 (28 hypotension group, 121 control group)	Chronic obstructive pulmonary disease (COPD), Sepsis, Other underlying diseases	Not specified (patients with underlying diseases)	Lidocaine (specific dosage/method not mentioned)	Lidocaine, low body weight, preintubation BP <140 mm Hg, and underlying conditions (e.g., COPD, sepsis) were significant factors associated with hypotension after RSI.	The presence of COPD and sepsis, low body weight, and lower preintubation BP were significant predictors of postintubation hypotension.	Clinical practitioners in the ED should take a patient's predisposing factors into serious consideration before emergency intubation while a preplanned strategy is made	Lidocaine usage was associated with postintubation hypotension.
2010	Tammy Abuan, Melissa Yeager, A. Bruce Montgomery [98]	Two double-blind, randomized, placebo-controlled clinical studies. Study 1: 12 weeks; Study 2: 20 weeks	Study 1-Mild/Moderate: 154 patients. Study 2-OCS: 114 patients	Asthma	Study 1-Mild/Moderate: Mild to moderate asthma. Study 2-OCS: More severe asthma with chronic oral corticosteroid (OCS) treatment.	Lidocaine solution for inhalation (LSI); 40 mg twice daily via eFlow nebulizer	- No improvement in pulmonary function (FEV1) in Study 1-Mild/Moderate after 12 weeks. - No corticosteroid-sparing effect in Study 2-OCS after 20 weeks.	No significant improvements in asthma symptom scores, peak expiratory flow values, FEV1 % predicted, asthma instability, or quality-of-life scores.	Inhaled lidocaine did not improve pulmonary function or reduce corticosteroid use in asthma treatment. It is not a useful treatment for asthma.	LSI was well tolerated. No specific side effects mentioned.
2013	Laura A Nafe, Vamsi P Guntur, John R Dodam, Tekla M Lee-Fowler, Leah A Cohn, Carol R Reinero [99]	Controlled Clinical Trial (2 weeks of treatment, 2-week washout)	14 cats (5 healthy, 9 asthmatic)	Feline Asthma	Experimental Asthma	Nebulized lidocaine (2 mg/kg q8h for 2 weeks)	Lidocaine did not alter BALF eosinophilia in healthy cats; in asthmatic cats, it increased EC200Raw (airway resistance)	EC200Raw increased significantly in asthmatic cats (10 ± 2 vs 5 ± 1 mg/ml, *P* = 0.043)	Lidocaine did not induce airway inflammation or hyper-responsiveness in healthy cats, but increased airway resistance in asthmatic cats	No specific side effects mentioned.
2018	F Tatulli, A Delcuratolo, A Caraglia, A Notarnicola, F P N Carbone, A Caputi [100]	Case report (duration: 90 minutes surgery)	1	Asthma	Partly-controlled asthma	Irrigation of the right subdiaphragmatic surface with lidocaine to control right shoulder pain	Three-trocar laparoscopic cholecystectomy under spinal anesthesia with low-pressure pneumoperitoneum (8 mmHg)	Successful surgery, no post-operative complications, discharge after 2 days	Spinal anesthesia and low-pressure pneumoperitoneum are safe and effective for patients with asthma in laparoscopic surgery	No specific side effects mentioned.
2022	Ananya Mahalingam-Dhingra, Melissa R. Mazan, Daniela Bedenice, Michelle Ceresia, Jill Minuto, Edward F. Deveney [101]	CONSORT-guided, randomized, double-blind, controlled pilot clinical trial, 14 days	19 recruited, 13 completed	Equine Asthma	Chronic/Active Asthma	Nebulized 1.0 mg/kg body weight q12h for 14 days	Lidocaine and budesonide cohorts both showed significant decreases in clinical scores. Lidocaine cohort showed significant reductions in BAL neutrophil percentage and tracheal mucus score. No significant changes in lung function.	Both groups showed significant clinical improvements (*P* < 0.05). Lidocaine significantly reduced BAL neutrophil percentage and tracheal mucus score. No changes in lung function parameters. No adverse events occurred.	Lidocaine may be a safe and effective treatment for equine asthma, particularly in horses intolerant to corticosteroids.	No specific side effects mentioned.

**Fig. 2:**
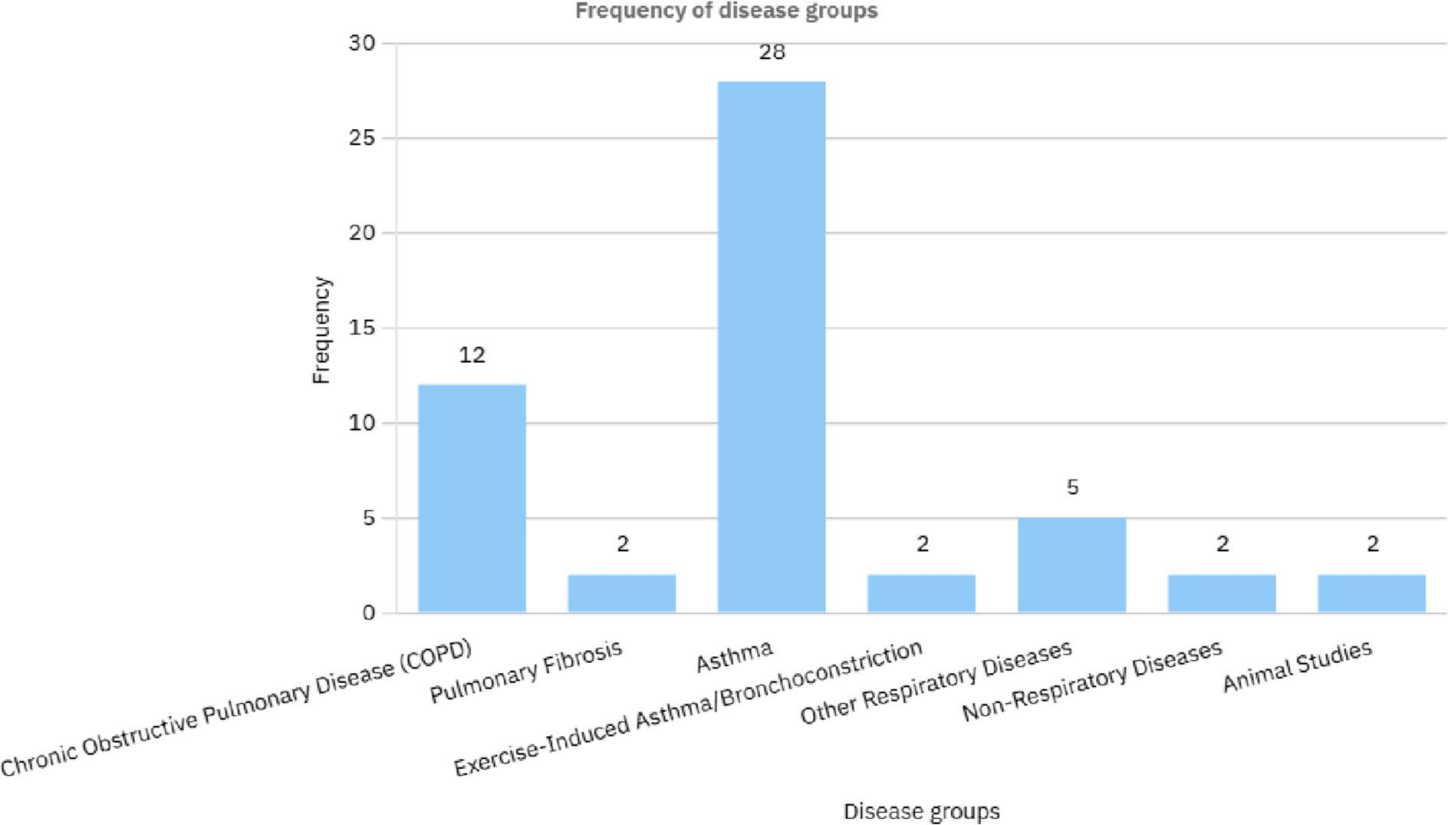
Frequency of diseases group types reviewed in Table 1

## Statistical analysis in the study of nebulized lidocaine for intractable cough in hospice care

### Data heterogeneity and the rationale for narrative synthesis

The studies included in this review presented a wide range of designs, such as randomized controlled trials (RCTs), observational studies, case reports, and case series. RCTs, known for their high—quality evidence, differed in patient groups, intervention details, and outcome measures. For example, one RCT might focus on a specific cancer patient subgroup, while another could target patients with a different stage of a respiratory disease. Observational studies provided real—world data but were prone to confounding factors. Case reports and case series, although valuable for unique cases, lacked the statistical power of larger studies. The outcome measures across these studies were equally diverse. Some studies used cough severity scores, others relied on pulmonary function tests like FEV1, and some focused-on patient—centered outcomes such as comfort and quality of life. Additionally, the dosing and administration of lidocaine varied greatly, with concentrations ranging from 1 to 4% and different frequencies of nebulization. This diversity made it difficult to conduct a traditional meta—analysis.

### Data extraction

To handle the diverse data, a standardized data extraction template was developed. This template covered various aspects of each study, including the author(s), publication year, study design, duration, number of participants, demographic details, disease condition and its stage, lidocaine dosage, administration method, and frequency. It also included key findings related to cough suppression, patient comfort, quality of life, reported outcomes (e.g., efficacy, adverse effects, patient tolerance), and side effects. For instance, data from Hunt et al.'s [55] study on the impact of nebulized lidocaine on oral glucocorticoid use were extracted following this template.

### Quality assessment different assessment tools were used based on the study design

*RCTs:* The Cochrane Risk of Bias Tool was utilized to evaluate RCTs. This tool assesses elements like randomization, which is crucial for ensuring comparable groups at the start of a trial and minimizing selection bias. Blinding, whether single—blind (where participants are unaware of treatment allocation) or double—blind (where both participants and researchers are unaware), helps reduce bias in outcomes. Reporting biases, which can occur when results are selectively reported, are also identified using this tool.

*Observational Studies:* The Newcastle—Ottawa Scale was employed to assess observational studies. It evaluates factors such as the selection of study participants, the comparability of groups, and the determination of outcomes. For example, it checks how well the study controls for confounding variables.

*Case Reports:* Case reports were evaluated according to the CARE (Case Report) guidelines. These guidelines ensure that case reports are well—structured, contain all relevant patient information, and clearly describe the intervention and its outcomes.

### Narrative synthesis

#### Synthesis of nebulized lidocaine research

Thematic analysis of extracted data identified two primary themes: efficacy in reducing cough severity and safety profiles across patient populations. Nebulized lidocaine demonstrated rapid cough alleviation (2–4 h) in cancer patients, while safety data highlighted common side effects like oropharyngeal numbness, bitter taste, and bronchoconstriction. Disease-specific outcomes revealed differential responses: in asthma patients classified by GINA criteria, lidocaine improved symptoms in mild-to-moderate persistent asthma (GINA Steps 2–3) but triggered bronchoconstriction in severe cases (Steps 4–5 [102, 103]). Mixed results emerged for COPD and pulmonary fibrosis, with some studies reporting symptom improvement and others no significant benefit or bronchoconstriction. These findings underscore the need for tailored lidocaine use based on disease severity and patient phenotype, particularly cautioning against its use in severe asthma due to broncho- constrictive risks.

#### Statistical findings and their interpretation

*Efficacy Variability:* The narrative synthesis revealed inconsistent results regarding the efficacy of nebulized lidocaine. In some studies, it effectively reduced cough frequency and severity, improved pulmonary function, and decreased glucocorticoid use. However, in other studies, especially those with different patient populations or dosing regimens, the results were less positive. For example, Abuan et al.'s 2010 study found that inhaled lidocaine did not improve pulmonary function or reduce corticosteroid use in asthma patients.

*Safety Profile:* Most studies reported a generally favorable safety profile for nebulized lidocaine, with many reporting no significant adverse effects. When side effects did occur, they were often mild and short—lived, such as oropharyngeal numbness and bitter taste. However, in certain patient groups like asthmatics, there was an increased risk of bronchoconstriction. McAlpine et al.'s 1989 study showed that 25% of asthmatic patients experienced a significant decline in FEV1 after lidocaine administration. In summary, the statistical analysis in this study was adapted to the complex and diverse nature of the available evidence. While the narrative synthesis provided useful insights, future research with more consistent study designs and larger sample sizes is needed to draw more conclusive results about the efficacy and safety of nebulized lidocaine in hospice care.

Lidocaine has been widely studied, with most research indicating a favorable safety profile. Numerous studies (e.g., [11, 55, 58, 59, 61, 63, 65, 67, 101]) reported no significant adverse effects, suggesting overall good tolerability. When side effects did occur, they were generally mild, including oropharyngeal numbness and a bitter taste [11]. Some cases of bronchoconstriction were noted, particularly in asthmatic patients [83], with 25% of subjects experiencing a fall in FEV1 exceeding 15%, reaching a maximum of 42.1%. A transient bronchospasm was also observed in a pediatric patient, resolving spontaneously within five minutes [96]. Although lidocaine is well-tolerated in most cases, certain populations exhibit an increased risk of adverse events. In asthmatic patients, several studies indicate that lidocaine can induce bronchoconstriction. For example, McAlpine et al. [83] reported significant airway constriction in 25% of patients following lidocaine administration. Similarly, Chang et al. [94] observed a 7% decrease in FEV1 and a 3% narrowing of airway diameter in asthmatic subjects. However, not all findings were negative. Nebulized lidocaine has demonstrated benefits in mild-to-moderate asthma cases, improving symptoms and reducing glucocorticoid use [93]. The impact of lidocaine on patients with COPD has also been evaluated. While it has been found to be generally safe for cough suppression [11] and procedural pain management [63, 64], concerns arise in cases of acute respiratory failure. In this population, lidocaine has been linked to worsened arterial blood gases (Murciano et al., 1982). Additionally, its use in intubated patients with COPD or sepsis has been associated with post-intubation hypotension. However, this effect was likely influenced by multiple factors, including pre-existing low blood pressure and body weight [97]. Beyond adult populations, lidocaine's safety in pediatric and pregnant patients has also been examined. It has been deemed safe in children with severe asthma [58], though a single case of transient bronchospasm was reported [96]. In pregnant patients, inhaled lidocaine was found to be effective for asthma management without adverse effects [62]. A closer look at statistical findings on safety and efficacy further supports these observations. In asthma patients, McAlpine et al. [83] reported that 25% (5 out of 20) experienced a > 15% fall in FEV1, with a maximum decline of 42.1%. Similarly, Chang et al. [94] found that IV lidocaine resulted in a 7% decrease in FEV1 (*P* = 0.006) and a 3% reduction in airway diameter (*P* < 0.001). On the other hand, Hunt et al. [93] demonstrated that nebulized lidocaine significantly improved FEV1 (*P* ≤ 0.001) and reduced nighttime awakenings (*P* ≤ 0.02), with no reported side effects. However, Abuan et al. [98] found that inhaled lidocaine was well tolerated but did not improve pulmonary function or corticosteroid use. In COPD and sepsis patients, Lin et al. [97] reported that among 149 patients, 28 (18.8%) experienced post-intubation hypotension, though lidocaine was not the sole contributing factor. These findings highlight the need for a nuanced approach to lidocaine administration. In asthma patients, its use should be approached with caution due to the risk of bronchoconstriction, although pretreatment with bronchodilators (e.g., salbutamol) may help mitigate this risk [89]. For COPD patients, lidocaine is safe for cough suppression and procedural analgesia but should be avoided in acute respiratory failure due to its impact on blood gases. In pediatric and pregnant patients, it appears to be generally well-tolerated, though close monitoring is recommended given the limited data available. Regardless of the patient population, monitoring for bronchoconstriction, hypotension, and airway narrowing is crucial, particularly in individuals with preexisting respiratory conditions. Furthermore, adherence to recommended dosages minimizes the risk of systemic toxicity, such as oropharyngeal numbness and altered taste perception (Refer to Table 1).

**Fig. 3:**
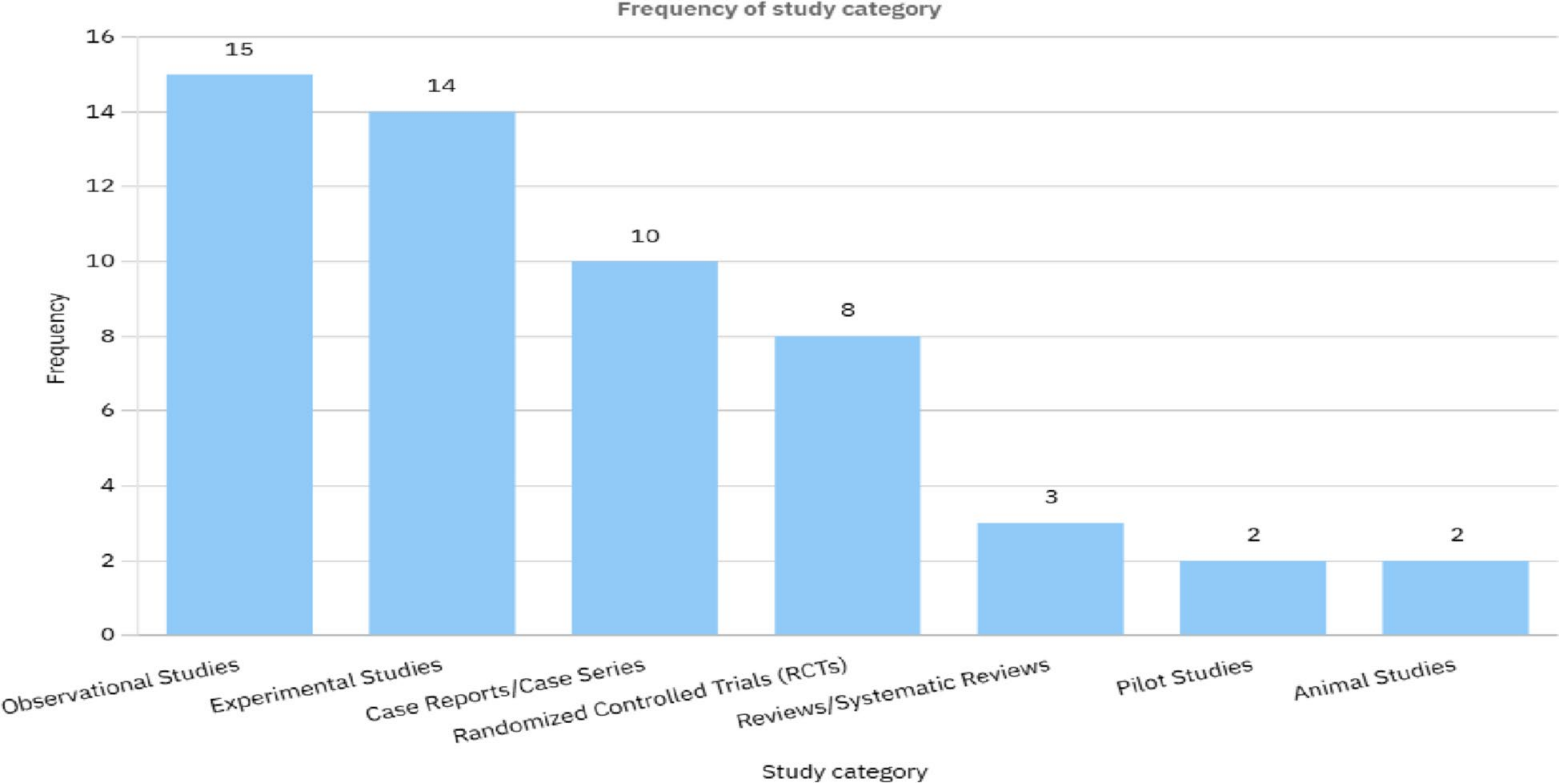
Frequency of study category reviewed in Table 1

**Fig. 4:**
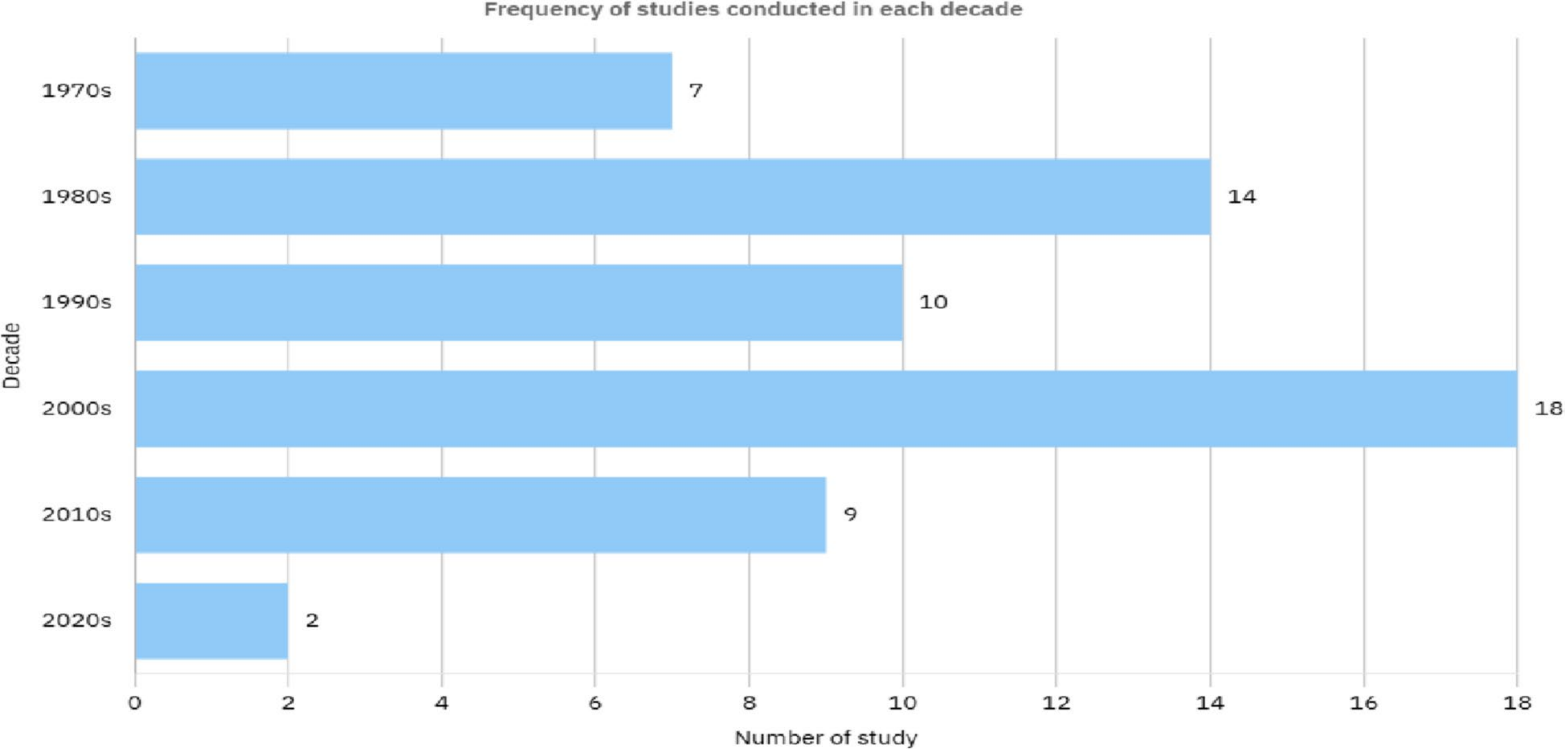
Frequency of number of Studies in each decade on Lidocaine usage for Cough in respiratory patients reviewed in Table 1

**Fig. 5:**
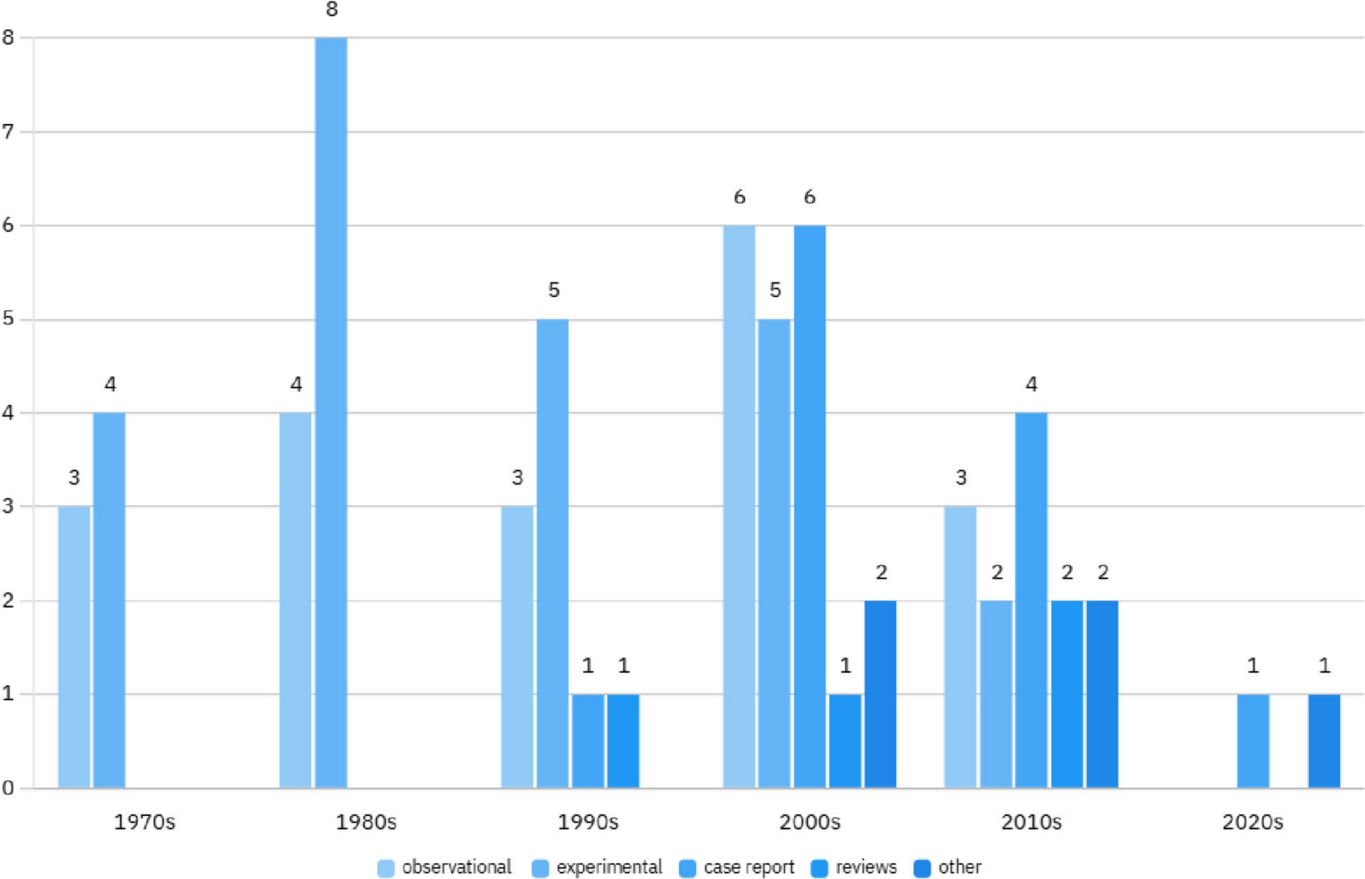
Frequency of study types in each decade on Lidocaine usage for Cough in respiratory patients reviewed in Table 1

In summary, lidocaine remains a valuable and effective medication, but its safety profile depends on the patient population and administration route. Careful patient selection, individualized treatment plans, and ongoing monitoring are essential to maximizing its benefits while minimizing potential risks. Figures 3, 4, and 5. A critical consideration not addressed in the reviewed studies is the potential role of preservatives in lidocaine solutions. Preservatives such as benzalkonium chloride, commonly found in multi-dose vials, are known to induce bronchoconstriction in sensitive populations, particularly asthmatics. This may confound the reported safety profile of nebulized lidocaine, as adverse events like bronchospasm (observed in 25% of asthmatic patients in McAlpine et al., 1989) could stem from preservative exposure rather than lidocaine itself. Notably, preservative-free lidocaine formulations are available and recommended for inhalational use to minimize airway irritation. However, none of the included studies explicitly detailed whether preservative-free solutions were utilized, limiting the ability to isolate lidocaine’s true safety profile. Clinicians should prioritize preservative-free formulations, especially in patients with airway hypersensitivity, to mitigate this risk.

### Practical considerations for nebulized lidocaine administration

Effective delivery of nebulized lidocaine requires attention to technique and patient education to optimize tolerability and adherence.

### Administration technique

#### Mouthpiece vs. mask for reduced oropharyngeal deposition

Using a mouthpiece positioned posteriorly in the throat minimizes contact with the tongue and oral mucosa, reducing the bitter taste and oropharyngeal numbness commonly reported with standard masks. This approach may enhance patient compliance, particularly in individuals sensitive to local anesthetic effects [104].

#### Post-nebulization oral rinsing ("Swish and Spit")

American Association for Respiratory Care (AARC) guidelines recommend rinsing the mouth after inhaled therapies to reduce residual drug exposure and local adverse effects. While focused on corticosteroids, this principle applies to lidocaine. Instructing patients to rinse their mouth with water immediately after nebulization ("swish and spit") reduces residual lidocaine in the oral cavity, mitigating dysgeusia (altered taste) and numbness [105].

#### Taste mitigation with mints/candy

Sucking on a sugar-free mint or candy post-administration can alleviate the unpleasant taste, improving patient experience. demonstrated that taste-masking strategies (e.g., mints, flavoring) improve adherence to inhaled therapies by reducing dysgeusia [106].

#### Transient initial cough exacerbation

Patients may experience a transient increase in cough during the first 1–2 min of nebulization due to airway irritation. Clinicians should forewarn patients about this phase, emphasizing the need to persist through it to achieve subsequent therapeutic benefit. Repeated dosing at regular intervals (e.g., every 2–4 h, aligned with lidocaine’s duration of action) avoids recurrent irritation, as residual anesthetic effects diminish gradually [9].

#### Dosing schedule

Regular administration before the prior dose fully wears off (e.g., within 2–4 h, depending on concentration) maintains therapeutic levels and minimizes the need to endure repeated initial cough exacerbations [107].

### Interpretation and clinical implications

Nebulized lidocaine has emerged as a promising therapeutic option for managing intractable cough in terminally ill patients receiving hospice care. Its localized action on the airway mucosa provides symptomatic relief without the systemic side effects commonly associated with opioids, such as sedation, constipation, and respiratory depression [29, 108]. This is particularly advantageous for frail patients who may already be experiencing polypharmacy or opioid-related complications. Unlike corticosteroids, which are often used for their anti-inflammatory properties, nebulized lidocaine does not carry the risk of immunosuppression, making it a safer alternative for patients with compromised immune systems [109, 110]. The efficacy of nebulized lidocaine in reducing cough frequency and severity has been demonstrated in various clinical settings, including asthma, chronic obstructive pulmonary disease (COPD), and post-operative cough suppression [111–113]. In hospice care, where the primary goal is to improve quality of life, the ability of nebulized lidocaine to provide rapid and effective relief from distressing cough symptoms is particularly valuable. Studies have shown that nebulized lidocaine can significantly reduce cough severity scores and improve patient comfort, often within minutes of administration [114, 115]. This rapid onset of action is crucial for terminally ill patients who may experience sudden and severe coughing episodes.

**Fig. 6:**
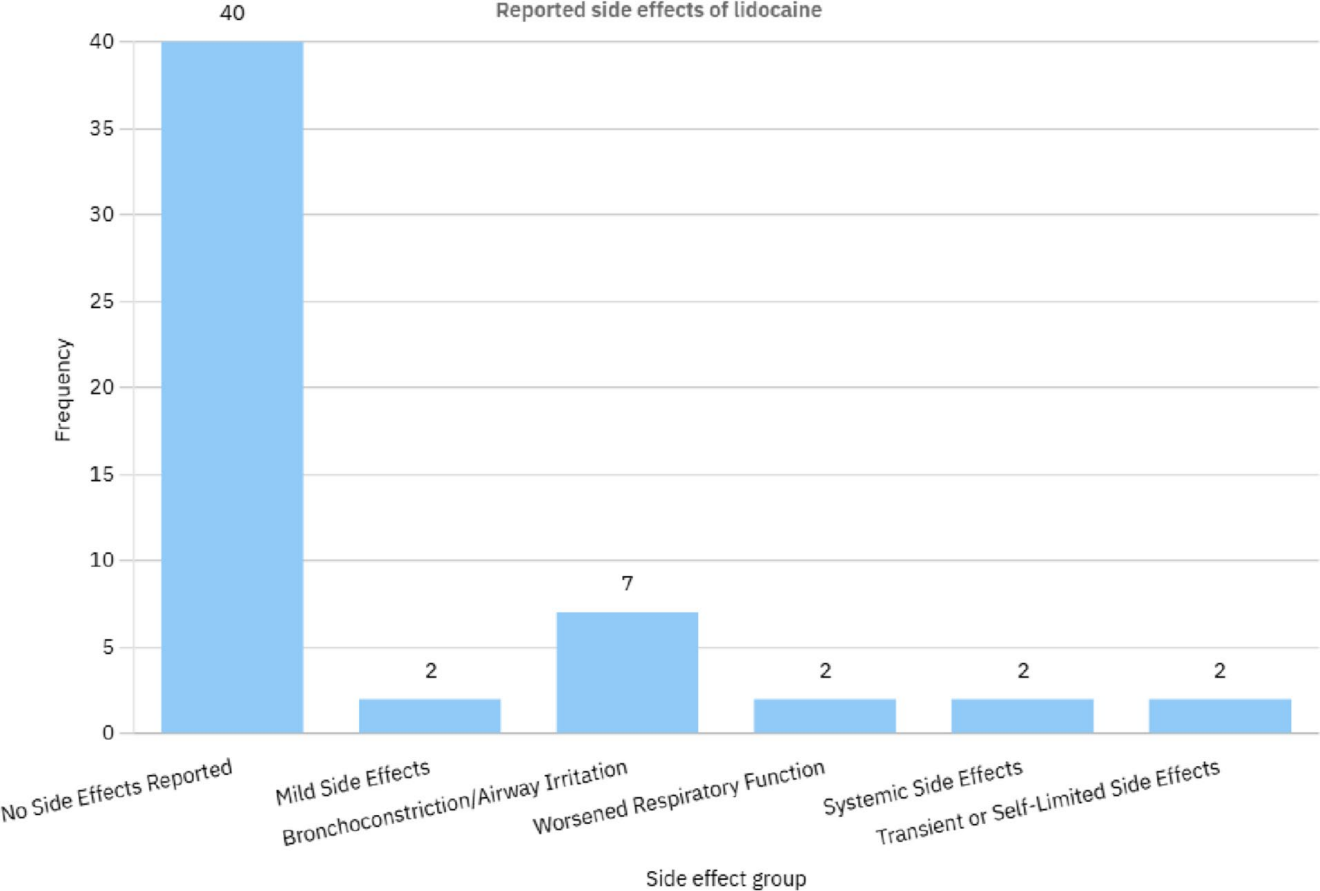
Frequency of reported side effects category of lidocaine usage for Cough in respiratory patients reviewed in Table 1

Moreover, nebulized lidocaine has been shown to be well-tolerated, with minimal side effects such as mild oropharyngeal numbness or a bitter taste, which are generally transient and do not require discontinuation of treatment [47, 116]. This favorable safety profile makes it a viable option for patients who may not tolerate other cough suppressants, such as opioids or antihistamines, due to their systemic effects. Figure 6, Table 2.

**Table 2:** Frequency of reported side effects category of lidocaine usage for Cough in respiratory patients and their examples reviewed in table

Side Effect Group		Frequency	Studies Included
No side effect reported		40	- Hunt et al. (1996) [55]- von Schönfeld et al. (1996)- M L Decco et al. (1999) [58] - K. Nozaki et al. (2001) [59] - C-F Chong et al. (2005) [11] - Shigekazu Sugino et al. (2006) [61] - Keiko Saito et al. (2006) [62] - Pyng Lee et al. (2007) [63] - Miroslava Kapala et al. (2007, 2009) [64]- Tammy Abuan et al. (2010) [98] - A. Molassiotis et al. (2010) [65] - K. Stuart-Smith et al. (2012)- Laura A Nafe et al. (2013) [99] - Naomi Ono et al. (2014) [67] - Casilda Olveira et al. (2014)- Adrian Sultana et al. (2017)- F Tatulli et al. (2018) [100] - Ananya Mahalingam-Dhingra et al. (2022) [101] - Yongbin Wang et al. (2023) [70]
Mild Side Effects		2	- C-F Chong et al. (2005) [11] (oropharyngeal numbness, bitter taste) - H Groeben et al. (2000) [90] (airway irritation)
**Bronchoconstriction/Airway Irritation**	7	- J E Fish et al. (1979)- L.G. McAlpine et al. (1989) [83] - H Groeben et al. (2000) [90] - Herng-Yu Sucie Chang et al. (2007) [94] - Burches et al. (2008) [96] - Chih-Chuan Lin et al. (2008) [97] - Laura A Nafe et al. (2013) [99]	
Worsened Respiratory Function	2	- D Murciano et al. (1982) (decreased PaO2, increased PaCO2) - Herng-Yu Sucie Chang et al. (2007) [94] (airway narrowing)	
Systemic Side Effects	2	- Chih-Chuan Lin et al. (2008) [97] (hypotension) - Burches et al. (2008) [96] (bronchospasm)	
**Transient or Self-Limited Side Effects**	2	- Burches et al. (2008) [96] (transient bronchospasm)- H Groeben et al. (2000) [90] (transient airway irritation)	

### Limitations of current evidence

Despite the promising findings, the current evidence supporting the use of nebulized lidocaine for intractable cough in hospice care is limited by several factors. First, the heterogeneity of study methodologies, including variations in dosing regimens, administration techniques, and outcome measures, makes it difficult to draw definitive conclusions about its efficacy [117, 118]. For example, some studies have used lidocaine concentrations ranging from 1 to 4%, with doses varying from 10 to 400 mg, leading to inconsistent results regarding optimal dosing [119, 120].

Second, many of the studies conducted to date have involved small sample sizes, which limits the generalizability of the findings. For instance, a study by Lim et al. [21] involving 99 patients reported significant improvements in cough severity, but the small cohort size raises questions about the reproducibility of these results in larger, more diverse populations. Additionally, the lack of large-scale randomized controlled trials (RCTs) specifically focused on hospice care settings further restricts the ability to establish robust evidence-based guidelines [2].Third, inconsistencies in reported efficacy have been observed, with some studies showing only partial relief of cough symptoms [121, 122]. This variability may be attributed to differences in patient populations, underlying etiologies of cough, and the presence of comorbid conditions. For example, patients with advanced lung cancer or COPD may respond differently to nebulized lidocaine compared to those with non-malignant causes of cough [123, 124].These discrepancies highlight the need for more standardized assessment tools and outcome measures to better evaluate the effectiveness of nebulized lidocaine. A significant constraint in this review is the inconsistent reporting of preservative content in lidocaine formulations across studies, which complicates the interpretation of safety outcomes such as bronchoconstriction. Without explicit details on preservative type and concentration, differentiating between lidocaine-related adverse effects and those caused by formulation additives remains challenging, potentially skewing risk assessments. Additionally, variability in administration techniques (e.g., mouthpiece vs. mask use, post-nebulization care protocols) among studies introduces ambiguity in comparing efficacy and tolerability. These methodological inconsistencies highlight the need for standardized reporting of formulation details and aerosol delivery practices in future research to enhance transparency and reproducibility. Such improvements would enable more precise evaluation of lidocaine’s therapeutic potential and safety profiles across diverse patient populations.

### Future research directions

To address these limitations, future research should focus on several key areas. First, there is a need for well-designed, large-scale RCTs to evaluate the efficacy and safety of nebulized lidocaine in hospice care settings. These trials should aim to establish standardized dosing regimens and administration protocols, as well as identify patient subgroups that are most likely to benefit from this treatment [11, 125].

Second, long-term safety profiles of nebulized lidocaine need to be established, particularly in patients with advanced illness who may require prolonged use. While short-term studies have shown that nebulized lidocaine is generally well-tolerated, the potential for cumulative side effects, such as systemic absorption leading to lidocaine toxicity, warrants further investigation [9, 126].

Third, future studies should incorporate patient-reported outcomes, such as quality of life measures, to better understand the impact of nebulized lidocaine on overall symptom burden and patient satisfaction. This is particularly important in hospice care, where the primary goal is to improve comfort and quality of life rather than to cure disease [127, 128]. Finally, research should explore the potential synergistic effects of combining nebulized lidocaine with other palliative therapies, such as opioids or corticosteroids, to enhance symptom control while minimizing side effects. For example, a study by Subedi et al. [33] demonstrated that the combination of lidocaine and dexamethasone was effective in reducing postoperative sore throat and cough, suggesting that similar combinations could be explored in hospice settings.

## Conclusion

Nebulized lidocaine offers a promising approach for managing intractable cough in hospice care, leveraging localized action and rapid symptom relief with minimal systemic impact, particularly beneficial for patient’s intolerant to opioids. However, evidence is constrained by methodological inconsistencies, small sample sizes, and limited randomized controlled trials. Future research should prioritize defining optimal dosages, evaluating long-term safety, and measuring patient-reported outcomes to establish clinical guidelines. Current practice requires tailored administration, considering patient tolerance, response, and symptom burden. Use of preservative-free formulations is critical to minimize broncho-constrictive risks, especially in asthma or COPD populations. Standardized reporting of lidocaine preparation details (e.g., preservative presence) in trials is essential to clarify safety profiles. To enhance utility, protocols should integrate patient-centered techniques such as mouthpiece delivery, post-treatment oral rinsing, and taste-masking strategies. Educating patients on transient cough exacerbation and structured dosing schedules may improve adherence. Prospective studies should systematically evaluate these practices to refine administration guidelines, ensuring safe and effective use of nebulized lidocaine in palliative care.

## Data Availability

No datasets were generated or analysed during the current study.
